# SPI-2/CrmA inhibits IFN-β induction by targeting TBK1/IKKε

**DOI:** 10.1038/s41598-017-11016-3

**Published:** 2017-09-05

**Authors:** Yue Qin, Mi Li, Sheng-Long Zhou, Wei Yin, Zhuan Bian, Hong-Bing Shu

**Affiliations:** 1 The State Key Laboratory Breeding Base of Basic Science of Stomatology, Hubei Province and Key Laboratory of Oral Biomedicine, Ministry of Education (Hubei-MOST KLOS & KLOBME), School and Hospital of Stomatology, Wuhan University, Wuhan, 430079 China; 2 State Key Laboratory of Virology, College of Life Sciences, Wuhan University, Wuhan, 430072 China; 3 Medical Research Institute, Collaborative Innovation Center for Viral Immunology, School of Medicine, Wuhan University, Wuhan, 430071 China

## Abstract

Viruses modulate the host immune system to evade host antiviral responses. The poxvirus proteins serine proteinase inhibitor 2 (SPI-2) and cytokine response modifier A (CrmA) are involved in multiple poxvirus evasion strategies. SPI-2 and CrmA target caspase-1 to prevent apoptosis and cytokine activation. Here, we identified SPI-2 and CrmA as negative regulators of virus-triggered induction of IFN-β. Ectopic expression of SPI-2 or CrmA inhibited virus-triggered induction of IFN-β and its downstream genes. Consistently, knockdown of SPI-2 by RNAi potentiated VACV-induced transcription of antiviral genes. Further studies revealed that SPI-2 and CrmA associated with TBK1 and IKKε to disrupt the MITA-TBK1/IKKε-IRF3 complex. These findings reveal a novel mechanism of SPI-2/CrmA-mediated poxvirus immune evasion.

## Introduction

Poxviruses comprise a large family of linear dsDNA viruses that replicate in the cytoplasm of host cells. The most widely studied genus, *Orthopoxviruse* (OPXV), is pathogenic to humans, cattle, and zoo animals. OPXVs include vaccinia virus (VACV), cowpox virus (CPXV), ectromelia virus (ECTV), and variola virus (VARV), the causative agent of smallpox. VACV, which was used in the vaccination campaign for smallpox eradication, is now being developed as live vaccines against other infectious diseases and cancer^1^. CPXV infects a wide range of host species and causes zoonosis, and the outbreaks of CPXV in recent years have caused a public health crisis^2^.

OPXVs have evolved various mechanisms to evade and suppress host antiviral responses^3^. They have large genomes with a variety of immunomodulatory genes and thus display a wide range of immune evasion strategies^4, 5^. Type I interferons (IFNs) are critical for both innate and adaptive immune responses against viral infection^6^. Accordingly, the suppression of type I IFN signaling by viral immunomodulatory proteins is one of the key events in virus immune evasion.

Host germline-encoded pattern-recognition receptors (PRRs) recognize viral nucleic acids and trigger downstream signaling events^7^. Viral DNA can be recognized by cyclic GMP-AMP synthase (cGAS)^8^, and viral RNA can be recognized by RIG-I-like receptors (RLRs)^7^. cGAS and RLRs activate the transcription factors interferon regulatory factor 3 (IRF3) and NF-κB though adaptor proteins, mediator of IRF3 activation (MITA, also termed STING) and virus-induced signaling adaptor (VISA, also termed MAVS), respectively^9–13^. The signaling pathways of IRF3 and NF-κB activation triggered by cGAS and RLRs converge at the level of TANK-binding kinase 1 (TBK1) and IκB kinases (IKKs). On the one hand, activation of cGAS or RLR leads to the recruitment of kinases (TBK1 and IKKε) by MITA or VISA^9–12^. MITA or VISA is phosphorylated by the kinases and subsequently recruits IRF3^14^. TBK1 and IKKε then phosphorylate IRF3, leading to the dimerization and nuclear translocation of IRF3^14^. On the other hand, inhibitor of NF-κB (IκB) is phosphorylated by the IKK complex (consisting of IKKα, IKKβ, and IKKγ) and then activated NF-κB is released^6^. Activated IRF3 and NF-κB respectively bind to interferon-stimulated response element (ISRE) and the κB site, leading to the transcriptional induction of IFN-β^7^.

Vaccinia virus (VACV) strain Western Reserve (WR) immediate-early gene *B13R* encodes a 38-kDa cytosolic protein SPI-2, which is highly homologous to its orthologue, CPXV cytokine response modifier A (CrmA). SPI-2 and CrmA are the most extensively studied OPXV serine protease inhibitors. They are non-essential for virus replication and are involved in multiple immunomodulatory events. It has been reported that SPI-2 and CrmA inhibit extrinsic apoptosis and host inflammatory responses^15–20^. SPI-2 and CrmA target caspase-1 to protect virus-infected cells from TNF- and Fas-mediated apoptosis as well as to prevent the proteolytic activation of interleukin-1β^16–19^.

In the present study, we found that SPI-2 and CrmA acted as inhibitors of both DNA- and RNA-virus-triggered induction of IFN-β. SPI-2 and CrmA functioned at the level of TBK1/IKKε to inhibit IRF3 but not NF-κB activation. SPI-2 and CrmA disrupted the MITA-TBK1/IKKε-IRF3 complex by interacting with TBK1 and IKKε. Our findings suggest that SPI-2 and CrmA antagonize the type I IFN pathway by targeting TBK1/IKKε, thus representing a newly identified mechanism of immune evasion of VACV and CPXV.

## Results

### Roles of SPI-2 orthologues in IFN-β induction

**Figure 1:**
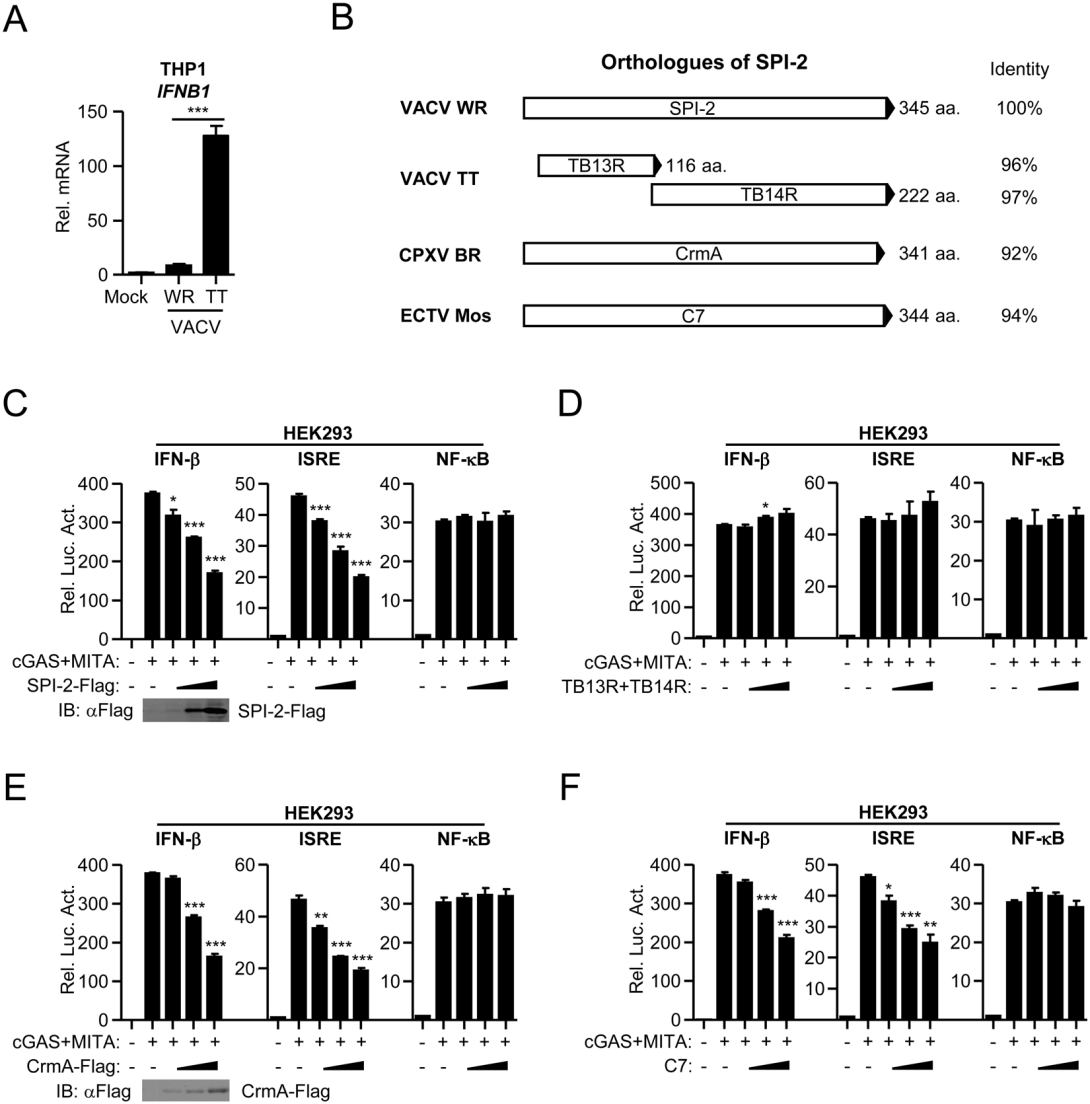
Roles of SPI-2 orthologues in IFN-β induction. (**A**) Transcription of IFN-β gene induced by VACV strains. THP-1 cells were infected with VACV strain WR or TT (MOI = 1) for 12 hours before quantitative real-time PCR analysis. (**B**) A schematic presentation of SPI-2 orthologues. aa., amino acid. (**C**–**F**) Effects of SPI-2 on cGAS-and-MITA-triggered activation of the IFN-β promoter, ISRE and NF-κB reporter. HEK293 cells were transfected with the indicated reporter, expression plasmids for cGAS and MITA, together with increasing amounts of SPI-2 (**C**), TB13R and TB14R (**D**), CrmA (**E**), and C7 (**F**) for 20 hours before the luciferase reporter assays. The lower blot shows the expression level of the transfected proteins.All experiments were repeated at least three times with similar results. The bar graphs show the mean ± S.D. (n = 3) of a representative experiment performed in triplicate. *p < 0.05, **p < 0.01, ***p < 0.001, relative to the control transfected with cGAS and MITA. The cropped blots are displayed, for full blots refer supplementary information.

VACV strain Tian-Tan (TT), a widely-used smallpox vaccine strain in China, is less virulent than strain WR^21, 22^. To evaluate the abilities of these VACV strains to stimulate type I IFN induction, THP-1 cells were infected with VACV strain WR and TT at an MOI of 1. Quantitative real-time PCR results indicated that both VACV strains triggered IFN-β induction in THP-1 cells. However, VACV strain TT activated IFN-β more than strain WR (Fig. 1A). In VACV strain WR and TT, several genes had polymorphic lengths, including *A39R*, *B13R*, *C4L*, *C14L*, and *C16R* ^22^. Among them, C4L and C16R have been demonstrated to regulate type I IFN induction^23, 24^. As type I IFN is critical in antiviral responses, we assumed that additional proteins target IFN induction signaling. SPI-2, encoded by the *B13R* gene in VACV strain WR, is split into two fragments (termed TB13R and TB14R) in VACV strain TT (Fig. 1B). As SPI-2 is highly homologous to CPXV CrmA, the well-known apoptosis inhibitor, we first constructed expression clones encoding VACV SPI-2, TB13R, and TB14R and identified their roles in IFN-β induction. Luciferase reporter assays were utilized to identify their abilities to regulate the activation of IFN-β promoter mediated by overexpression of cGAS and MITA in HEK293 cells. Ectopic expression of SPI-2 inhibited cGAS-and-MITA-mediated activation of IFN-β promoter and ISRE reporter, but not NF-κB reporter, in a dose-dependent manner (Fig. 1C). However, ectopic expression of TB13R together with TB14R failed to inhibit cGAS-and-MITA-mediated activation of IFN-β promoter and ISRE reporter (Fig. 1D). Although the sequences of TB13R and TB14R are highly homologous to SPI-2, these two proteins do not retain the function of the full-length protein, which may be due to the conformational change resulting from splitting ORFs. These data indicated that full-length SPI-2 inhibits cGAS-and-MITA-triggered IFN-β induction.

Next, we identified the roles of SPI-2 orthologues in IFN-β induction. CPXV CrmA and ECTV C7 proteins have 92% and 94% amino acid identity with SPI-2 protein, respectively (Fig. 1B). Ectopic expression of CrmA and C7 inhibited cGAS-and-MITA-mediated activation of IFN-β promoter and ISRE reporter, but not NF-κB reporter, in a dose-dependent manner (Fig. 1E and F). Thus, the SPI-2 orthologues, CrmA and C7, inhibit cGAS-and-MITA-triggered IFN-β induction.

Sendai virus (SeV) triggers induction of type I IFNs through RLR signaling. Consistent with their roles in regulation of cGAS-and-MITA-mediated activation of IFN-β promoter, ectopic expression of SPI-2, CrmA, and C7, but not TB13R and TB14R, inhibited SeV-induced activation of IFN-β promoter and ISRE reporter but not NF-κB reporter (Supplementary Fig. S1A to D). They also had no marked effect on IFN-γ-induced activation of IRF1 promoter (Supplementary Fig. S1E to H). These data suggest that SPI-2 and its homologues, CrmA and C7, inhibit SeV-induced activation of IFN-β promoter.

### SPI-2 and CrmA inhibit virus-triggered induction of endogenous IFN-β

**Figure 2:**
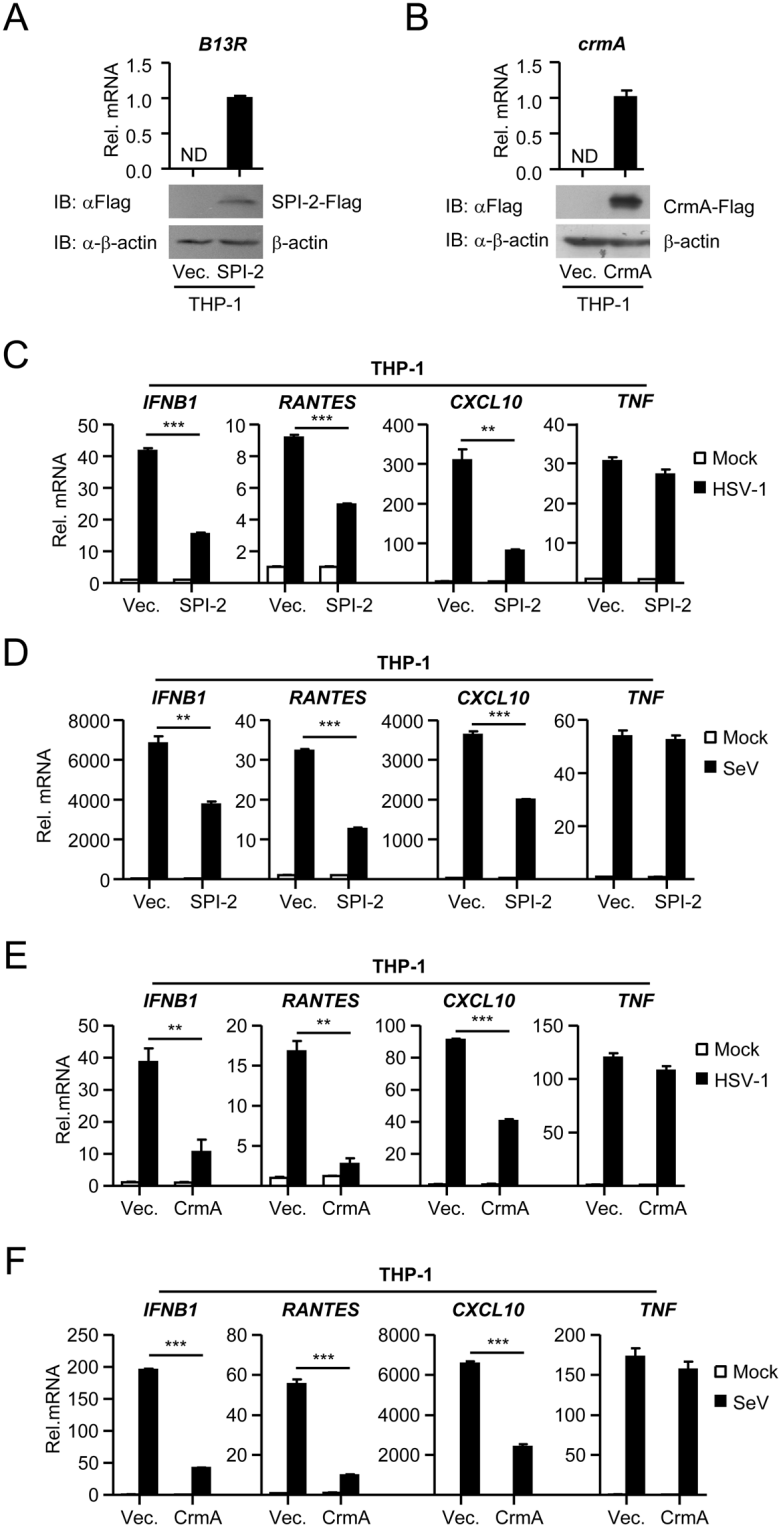
Effects of SPI-2 and CrmA on virus-triggered induction of endogenous IFN-β. (**A**,**B**) Quantitative real-time PCR and immunoblot analysis of SPI-2 and CrmA expression in THP-1 cells stably transfected with SPI-2-Flag or CrmA-Flag plasmid. SPI-2-Flag (**A**) and CrmA-Flag (**B**) stable THP-1 cells were analyzed by quantitative real-time PCR and immunoblots. ND, not detected. (**C**,**D**) Effects of SPI-2 on HSV-1- and SeV-induced transcription of antiviral genes. SPI-2-Flag stable THP-1 cells were infected with HSV-1 (**C**) or SeV (**D**) for 12 hours before quantitative real-time PCR analysis. (**E**,**F**) Effects of CrmA on HSV-1- and SeV-induced transcription of antiviral genes. The experiments were performed similar to those in (**C**,**D**). All experiments were repeated at least three times with similar results. The bar graphs show the mean ± S.D. (n = 3) of a representative experiment performed in triplicate. *p < 0.05, **p < 0.01, ***p < 0.001. The cropped blots from different gels are displayed, for full blots refer supplementary information.

To investigate the roles of SPI-2 and CrmA in virus-triggered induction of endogenous IFN-β, THP-1 cells were stably transfected with Flag-tagged SPI-2 or CrmA (Fig. 2A and B). Quantitative real-time PCR analysis indicated that ectopic expression of SPI-2 inhibited herpes simplex virus 1 (HSV-1)- and SeV-induced transcription of *IFNB1* and its downstream genes *RANTES* and *CXCL10*, but not *TNF*, downstream of NF-κB signaling (Fig. 2C and D). Similarly, CrmA inhibited HSV-1- and SeV-induced transcription of *IFNB1*, *RANTES*, and *CXCL10*, but not *TNF* (Fig. 2E and F). These data suggest that SPI-2 and CrmA specifically inhibit virus-triggered induction of endogenous IFN-β.

Since SPI-2 and CrmA inhibited virus-triggered induction of IFN-β, we examined their roles in cellular antiviral responses. As shown in Supplementary Fig. S2, HSV-1 and Vesicular Stomatitis Virus (VSV) production was increased in THP-1 cells ectopically expressing SPI-2 or CrmA, consistent with the role of SPI-2 and CrmA in negative regulation of both DNA- and RNA-virus-triggered IFN-β induction. These results suggest that SPI-2 and CrmA negatively regulate cellular antiviral responses.

### Knockdown of SPI-2 enhances VACV-triggered induction of IFN-β gene

**Figure 3:**
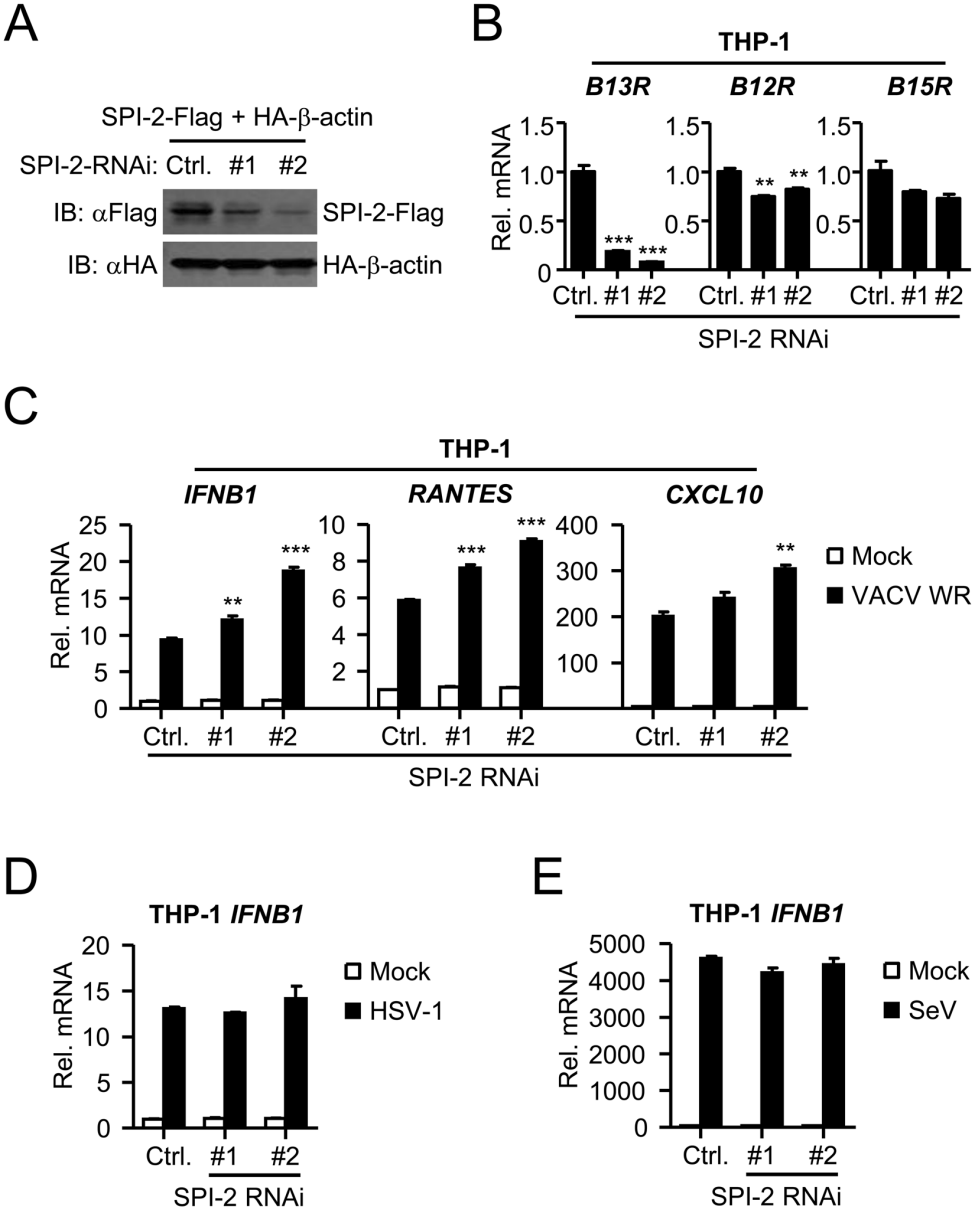
Knockdown of SPI-2 enhances VACV-triggered induction of IFN-β. (**A**) Effects of SPI-2-RNAi plasmids on the expression of SPI-2. HEK293 cells were transfected with the indicated plasmids for 24 hours before immunoblot analysis. (**B**) Effects of SPI-2-RNAi plasmids on the transcription of *B13R* and neighboring genes. SPI-2-RNAi stable THP-1 cells were infected with VACV strain WR for 12 hours before quantitative real-time PCR analysis. (**C**) Effects of SPI-2-RNAi plasmids on VACV-induced transcription of antiviral genes. SPI-2-RNAi stable THP-1 cells were infected with VACV strain WR for 12 hours before quantitative real-time PCR analysis. *p < 0.05, **p < 0.01, ***p < 0.001, relative to the control with VACV WR infection. (**D**,**E**) Effects of SPI-2-RNAi plasmids on HSV-1- and SeV-induced transcription of *IFNB1*. SPI-2-RNAi stable THP-1 cells were infected with HSV-1 (**D**) or SeV (**E**) for 12 hours before quantitative real-time PCR analysis.All experiments were repeated at least three times with similar results. The bar graphs show the mean ± S.D. (n = 3) of a representative experiment performed in triplicate. The cropped blots from different gels are displayed, for full blots refer supplementary information.

The role of endogenous SPI-2 in innate antiviral responses were next examined. RNAi knockdown strategy used in our study has been successfully used in previous studies^25, 26^. Two SPI-2-RNAi plasmids were constructed that could inhibit the mRNA level of *B13R* gene (Fig. 3A and B). In THP-1 cells stably transfected with the SPI-2-RNAi plasmids, the mRNA levels of *B13R* neighboring genes, *B12R* and *B15R*, were not dramatically decreased (Fig. 3B). Quantitative real-time PCR analysis indicated that knockdown of SPI-2 promoted VACV-induced transcription of *IFNB1*, *RANTES* and *CXCL10* (Fig. 3C), but it had no marked effect on HSV-1- or SeV-induced transcription of *IFNB1* (Fig. 3D and E). These results suggest that knockdown of SPI-2 potentiates VACV-triggered induction of IFN-β and its downstream genes.

### SPI-2 and CrmA inhibit IRF3 activation at the level of TBK1/IKKε

**Figure 4:**
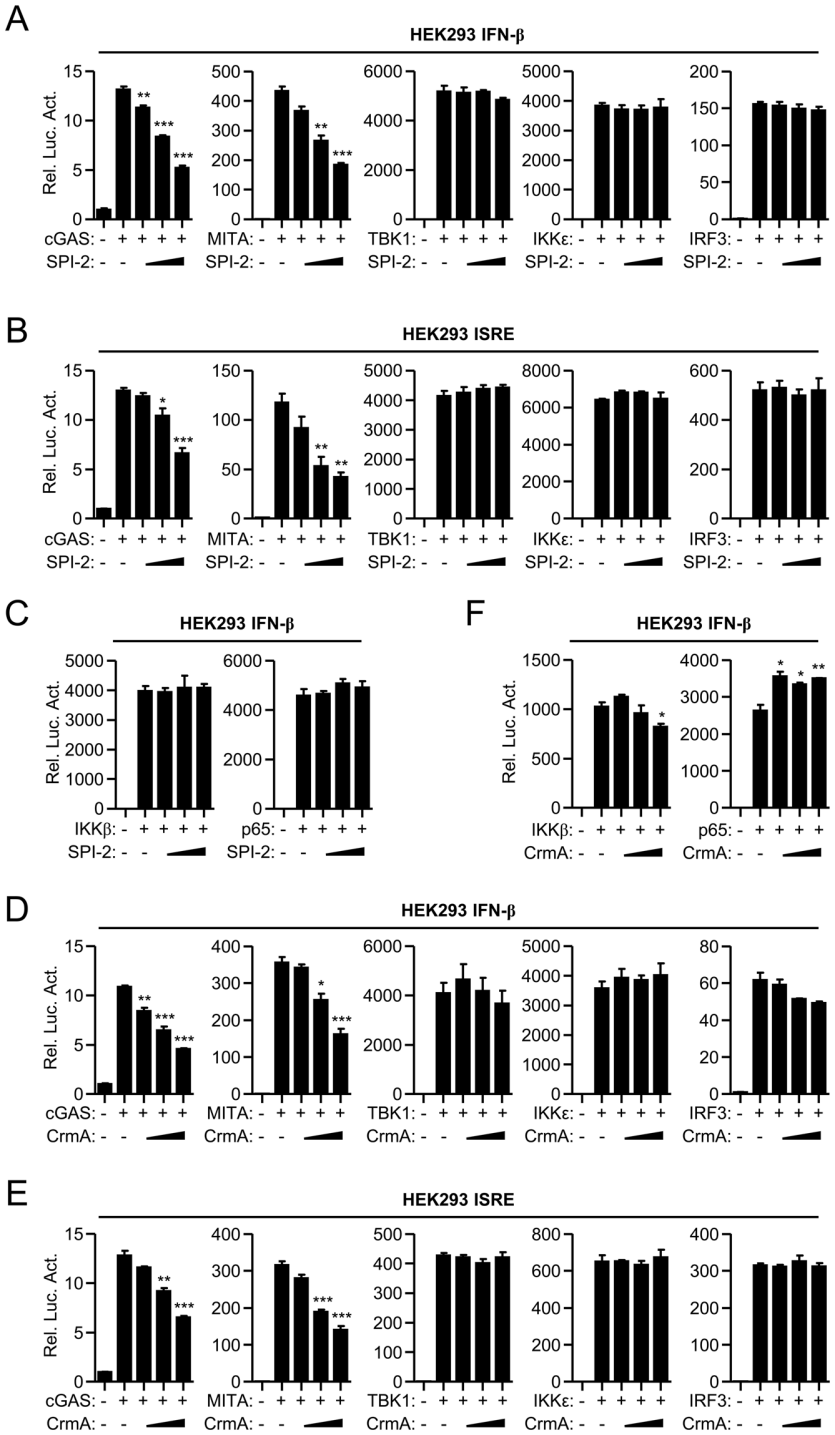
Effects of SPI-2 and CrmA on IFN-β promoter activation mediated by various components. (**A**,**B**) Effects of SPI-2 on IFN-β promoter (**A**) and ISRE reporter (**B**) activation mediated by cGAS, MITA, TBK1, IKKε, and IRF3. HEK293 cells were transfected with the IFN-β promoter or ISRE reporter and the indicated expression plasmids together with increasing amounts of SPI-2 for 20 hours before the luciferase reporter assays. (**C**) Effects of SPI-2 on IKKβ- and p65-mediated activation of IFN-β promoter. HEK293 cells (2 × 10^5^) were transfected with the IFN-β promoter (0.1 μg) and IKKβ or p65 (0.1 μg each) together with increasing amounts of SPI-2 (25, 50, 100 ng) for 20 hours before the luciferase reporter assays. (**D**,**E**) Effects of CrmA on IFN-β promoter (**D**) and ISRE reporter (**E**) activation mediated by cGAS, MITA, TBK1, IKKε, and IRF3. The experiments were performed similarly to those in (**A**,**B**). (**F**) Effects of CrmA on IKKβ- and p65-mediated activation of IFN-β promoter. The experiments were performed similarly to those in (**C**). All experiments were repeated at least three times with similar results. The bar graphs show the mean ± S.D. (n = 3) of a representative experiment performed in triplicate. *p < 0.05, **p < 0.01, ***p < 0.001, relative to the controls transfected with the indicated components.

To determine which step of IFN-β induction was targeted by SPI-2/CrmA, luciferase assays was utilized in which SPI-2 or CrmA was co-expressed with the components of IFN-β induction signaling. Ectopic expression of SPI-2 inhibited the activation of IFN-β promoter and ISRE reporter mediated by overexpression of cGAS and MITA but not their downstream TBK1, IKKε, and IRF3 (Fig. 4A and B). IKKβ and p65 act upstream of NF-κB activation. Consistent with the role of SPI-2 in NF-κB activation (Figs 1C, 2C and D), SPI-2 did not markedly affect IKKβ- or p65-mediated activation of IFN-β promoter (Fig. 4C). Similarly, CrmA inhibited cGAS-, MITA-, but not TBK1-, IKKε- and IRF3-mediated activation of IFN-β promoter and ISRE reporter (Fig. 4D and E). Additionally, CrmA had no marked effect on IKKβ- or p65-mediated activation of IFN-β promoter (Fig. 4F). SPI-2 and CrmA also inhibited the activation of IFN-β promoter and ISRE reporter mediated by the RLR adaptor VISA, which is upstream of TBK1/IKKε (Supplementary Fig. S3). These data indicate that SPI-2 and CrmA inhibit IRF3 activation at the level of TBK1/IKKε.

### SPI-2 and CrmA disrupt the MITA-TBK1/IKKε-IRF3 complex by interacting with TBK1/IKKε

**Figure 5:**
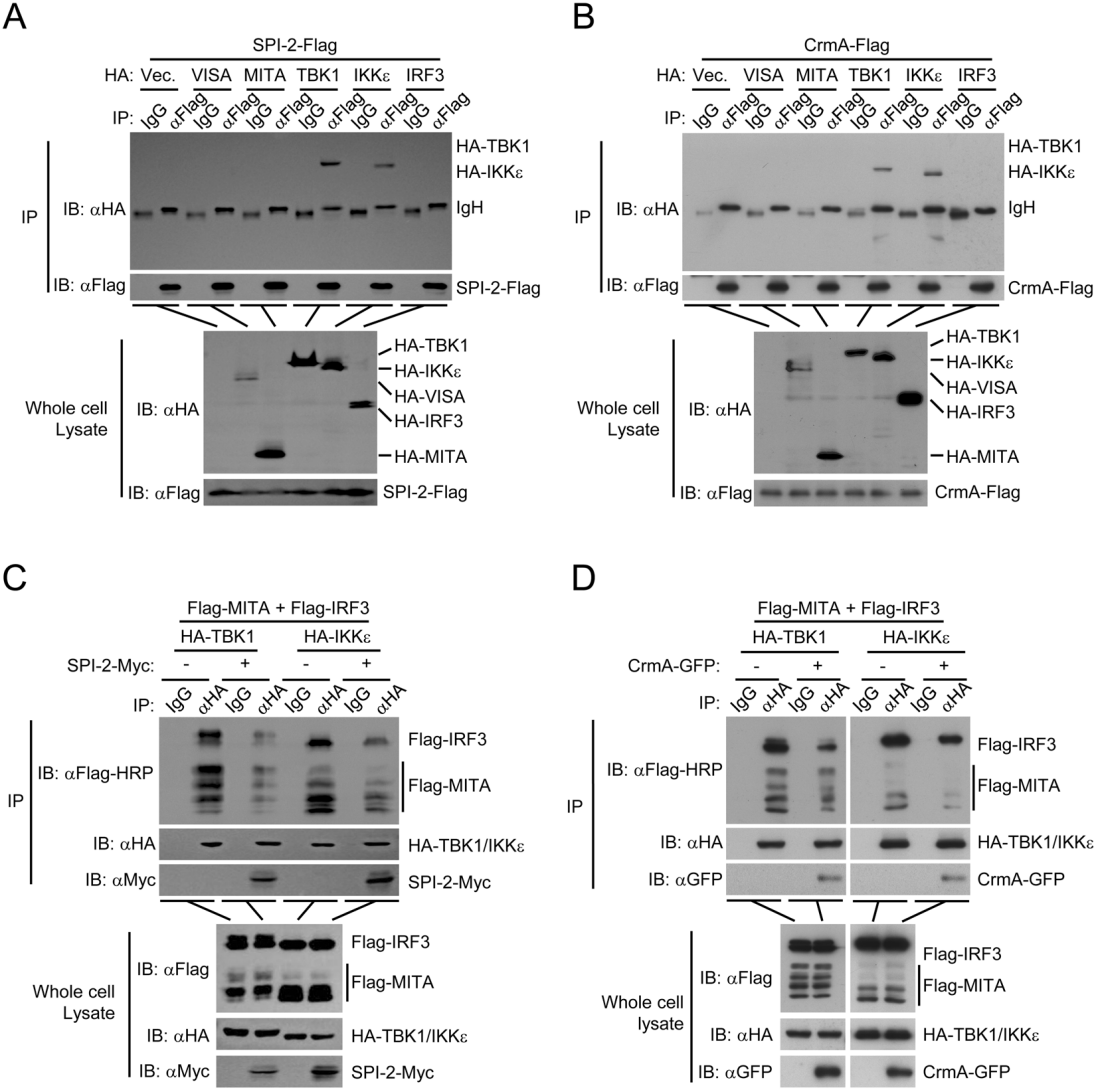
SPI-2 and CrmA disrupt the MITA-TBK1/IKKε-IRF3 complex. (**A**, **B**) Association of SPI-2 (**A**) and CrmA (**B**) with TBK1 and IKKε. HEK293 cells were transfected with the indicated plasmids for 24 hours before coimmunoprecipitation and immunoblot analysis with the indicated antibodies.(**C**,**D**) SPI-2 (**C**) and CrmA (**D**) disrupt the MITA-TBK1/IKKε-IRF3 complex. HEK293 cells were transfected with the indicated plasmids for 20 hours before coimmunoprecipitation and immunoblot analysis with the indicated antibodies. All experiments were repeated at least three times with similar results. The cropped blots from different gels are displayed, for full blots refer supplementary information.

Since SPI-2 and CrmA function at the level of TBK1/IKKε, we examined whether SPI-2 and CrmA interacted with TBK1 and IKKε. Transient transfection and co-immunoprecipitation experiment indicated that SPI-2 and CrmA were associated with TBK1 and IKKε in HEK293 cells (Fig. 5A and B). To investigate the mechanism by which SPI-2 and CrmA regulated IRF3 activation, we examined whether SPI-2 and CrmA affected the interaction between components of the MITA-TBK1/IKKε-IRF3 complex as well as the stability of the components. Ectopic expression of SPI-2 and CrmA attenuated the interaction of TBK1/IKKε with MITA and IRF3, and SPI-2 and CrmA did not markedly affect the protein levels of MITA, TBK1, IKKε, and IRF3 (Fig. 5C and D). These results suggested that SPI-2 and CrmA disrupted the MITA-TBK1/IKKε-IRF3 complex through interacting with TBK1 and IKKε. Thus, SPI-2/CrmA inhibits IFN-β induction by targeting TBK1/IKKε.

## Discussion

Previous studies have demonstrated that SPI-2 and CrmA have anti-apoptotic and anti-inflammatory functions^15–20^. Here, we identified SPI-2 and CrmA as negative regulators of virus-triggered induction of IFN-β. Our findings reveal a novel mechanism of SPI-2/CrmA mediated OPXV immune evasion.

**Figure 6:**
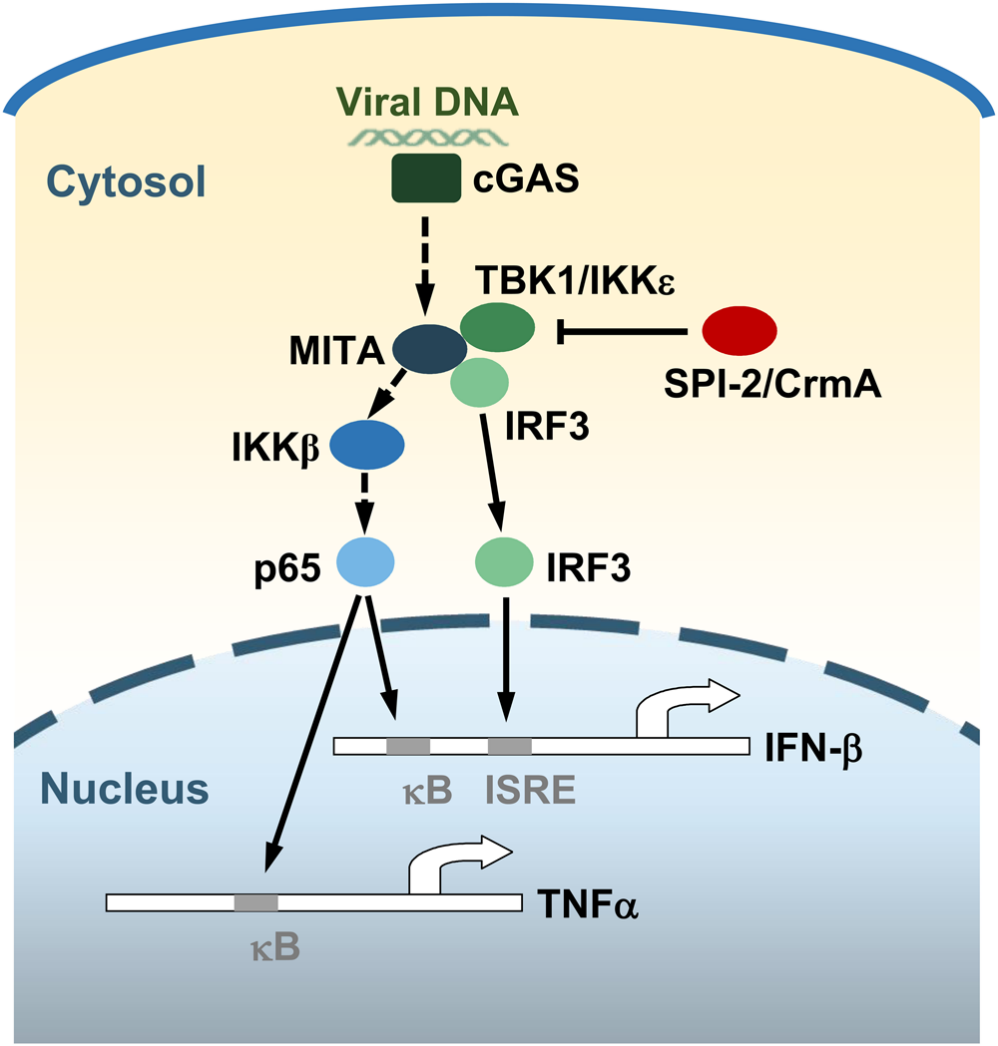
A model of the roles of SPI-2/CrmA in virus-triggered induction of downstream genes. Recognition of cytosolic viral DNA by cGAS triggers the activation of MITA. Activated MITA in turn activates IRF3 and NF-κB to trigger the induction of type I IFNs through TBK1/IKKε and IKKβ, respectively. The poxvirus proteins SPI-2 and CrmA inhibit the induction of type I IFNs by targeting TBK1 and IKKε.

SPI-2 and its orthologue CrmA inhibited the induction of IFN-β triggered by both DNA and RNA viruses, and SPI-2 and CrmA inhibited the activation of IFN-β promoter triggered by cGAS and RLR signaling. SPI-2 and CrmA specifically inhibited virus-triggered induction of IFN-β by inhibiting of IRF3, but not NF-κB, activation. Luciferase reporter assays suggested that SPI-2 and CrmA inhibited the activation of IFN-β promoter and ISRE reporter at the level of TBK1 and IKKε, consistent with the roles of TBK1 and IKKε in the activation of IRF3 but not NF-κB. Further experiments suggested that SPI-2 and CrmA were associated with TBK1 and IKKε. Additionally, TBK1 and IKKε disrupted the MITA-TBK1/IKKε-IRF3 complex. Based on our findings, we developed a working model of how SPI-2 and CrmA negatively regulate virus-triggered type I IFN induction (Fig. 6). The recognition of cytosolic viral DNA by PRRs triggers host antiviral responses. SPI-2 and CrmA inhibit the induction of type I IFNs by targeting TBK1 and IKKε. These findings have not only verified a novel immunomodulatory function of SPI-2/CrmA, but they also provide an example of how viruses escape host immune attack using distinct mechanisms mediated by one immunomodulator.

Consistent with the ability of SPI-2 to inhibit IFN-β induction, knockdown of SPI-2 by RNAi enhanced VACV-induced transcriptional activation of IFN-β gene in THP-1 cells. We observed that knockdown of *B13R* gene by SPI-2-RNAi plasmids slightly inhibited the mRNA levels of its neighboring genes, *B12R* and *B15R*, likely because the elevation of IFN-β production inhibited the replication of VACV in SPI-2 knockdown cells.

Multiple viral proteins may target a same cellular pathway to ensure successful immune evasion^3^. VACV encodes several immunomodulatory proteins targeting type I IFNs induction signaling, including E3^27, 28^, A46^29, 30^, C16^24^, K7^31^, C6^32^, and N2^33^. Among them, K7 and C6 have been reported to inhibit IRF3 activation by targeting the kinase complex containing TBK1 and IKKε. However, each protein targets distinct components of this complex. K7 inhibits IRF3 phosphorylation by targeting DDX3^31^, and C6 interacts with TANK, NAP1, and SINTBAD, the scaffold proteins that associate with TBK1 and IKKε^32^. Our findings suggest that SPI-2 inhibits the activation of IRF3 in a different manner than K7 and C6. SPI-2 directly targets the kinases TBK1 and IKKε, which are responsible for the phosphorylation of both MITA and IRF3. SPI-2 may work in coordination with these immunomodulatory proteins to inhibit the activation of IRF3 triggered by VACV infection.

The mechanism study of immune evasion mediated by SPI-2 and CrmA not only reveals new insights into the virus-host interaction but also has important implications for rational vaccine design and antiviral drug development against OPXV infection.

## Materials and Methods

### Reagents and antibodies

Recombinant IFN-γ (R&D Systems) and mouse monoclonal antibodies against FLAG, Flag-HRP, β-actin (Sigma), Myc (CST), and HA (Covance) were purchased from the indicated manufacturers. HEK293 and THP-1 cells, SeV, HSV-1, and VSV have been previously described^26, 34–36^.

### Constructs

IFN-β, ISRE, NF-κB and IRF1 luciferase reporter plasmids, mammalian expression plasmids for Flag- or HA-tagged cGAS, MITA, TBK1, IKKε, IRF3, and β-actin have been previously described^26, 34^. Mammalian expression plasmids for human Flag-, HA- or Myc-tagged SPI-2 and CrmA were constructed using standard molecular biology techniques.

### RNAi

Double-stranded oligonucleotides corresponding to the target sequences were cloned into the pSuper.Retro-RNAi plasmid (Oligoengine). The following sequences were targeted for SPI-2 mRNA: #1 5′-GGAGAACATGGATAAGGTT-3′, #2 5′-GCGATATTCTGCCGTGTTT-3′. The sequence targeted by the control RNAi plasmid is 5′-GGAAGATGTATGGAGACATGG-3′.

### Transfection and reporter assays

HEK293 cells were seeded and transfected the following day using the standard calcium phosphate precipitation method or FuGENE (Roche), according to the procedures recommended by the manufacturer. Empty control plasmids were added to ensure that each transfection received the same amount of total DNA. To normalize for transfection efficiency, pRL-TK Renilla luciferase reporter plasmids were added to each transfection. Luciferase assays were performed using a dual-specific luciferase assay kit (Promega). Firefly luciferase activities were normalized based on Renilla luciferase activities.

### Quantitative real-time PCR

Total RNA was isolated from THP-1 cells (1 × 10^6^) using TRIzol reagent (Takara) according to the manufacturer’s instructions. cDNA was synthesized from 2 μg of purified total RNA using reverse transcriptase (Invitrogen). Quantitative real-time PCR was performed using Ssoadvanced Universal SYBR Green Supermix (Bio-Rad) to measure the expression of mRNA. The related fold difference in expression of the target gen between the cells were analyzed with 2^∆∆CT^ method. Data represent the relative abundance of the indicated mRNA normalized to that of GAPDH. Gene-specific primer sequences were as described^34^ or as follows: B13R: (forward, 5′-CTCCGACGGAAATGGTAGAT-3′; reverse, 5′-TGCCGAATGATTCCTTTACA-3′). CrmA: (forward, 5′-GTGTCGACGCTATGATCCAC-3′; reverse, 5′-GATGAACGGATGATCTGCAC-3′). B12R: (forward, 5′-ACAACTGGACACGGGAACG-3′; reverse, 5′-CCTTTGGGGCGAATACTCTT-3′). B15R: (forward, 5′-TTAACGCGCCTGAATGTATCG-3′; reverse, 5′-CAGTAGGTTTCGTTCGTGGTAATG-3′).

### RNAi-transduced stable THP-1 cells

HEK293 cells were transfected with two packaging plasmids (pGAG-Pol and pVSV-G) together with control or SPI-2-RNAi retroviral plasmids respectively by calcium phosphate precipitation. Twenty-four hours after transfection, the cells were incubated with new medium without antibiotics for another twenty-four hours. The recombinant virus-containing medium was filtered with a 0.22-μm filter (Millex) and then added to cultured THP-1 cells in the presence of polybrene (4 μg/mL). The infected cells were selected with puromycin (0.5 μg/mL) for at least seven days before performing additional experiments.

### Coimmunoprecipitation and immunoblot analysis

HEK293 cells (5 × 10^6^) were lysed in 1 mL IP lysis buffer (20 mM Tris-HCl [pH 7.4], 150 mM NaCl, 1 mM EDTA, 1% Triton X-100, 10 μg/mL aprotinin, 10 μg/mL leupeptin, and 1 mM phenylmethylsulphonyl fluoride) on wet ice. Coimmunoprecipitation and immunoblot analysis were performed as previously described^10, 26, 34–36^.

### Data Availability

All data generated or analyzed during this study are included in this published article (and its Supplementary Information files).

## Electronic supplementary material

Dataset 1
