# Supplementary material for: SPI-2/CrmA inhibits IFN-β induction by targeting TBK1/IKKε
Source: Sci Rep. 2017 Sep 5;7:10495. doi: 10.1038/s41598-017-11016-3 (PMC5585206; doi:10.1038/s41598-017-11016-3)

**SPI-2/CrmA inhibits IFN-β induction by targeting TBK1/IKKε.**

Yue Qin^1^*, Mi Li^2^, Sheng-Long Zhou^1^, Wei Yin^1^, Zhuan Bian^1^, Hong-Bing Shu^2,3^.

^1^ The State Key Laboratory Breeding Base of Basic Science of Stomatology, Hubei province and Key Laboratory of Oral Biomedicine, Ministry of Education (Hubei-MOST KLOS & KLOBME), School and Hospital of Stomatology, Wuhan University, Wuhan, China 430079

^2^ State Key Laboratory of Virology, College of Life Sciences, Wuhan University, Wuhan, China 430072

^3^ Medical Research Institute, Collaborative Innovation Center for Viral Immunology, School of Medicine, Wuhan University, Wuhan, China 430071

**Supplementary Dataset**

**Figure S1. Roles of SPI-2 orthologues in SeV-induced activation of IFN-β promoter and IFN-γ-induced activation of IRF-1 promoter.**

(A to D) Effects of SPI-2 and its orthologues on SeV-induced activation of the IFN-β promoter, ISRE and NF-κB reporter. HEK293 cells were transfected with the indicated reporter together with increasing amounts of SPI-2 (A), CrmA (B), TB13R and TB14R (C), and C7 (D). Twenty hours after transfection, the cells were infected with SeV for 12 hours or left uninfected before the luciferase reporter assays.

(E to H) Effects of SPI-2 and CrmA on IFN-γ-induced activation of IRF-1 promoter. HEK293 cells were transfected with the IRF-1 promoter reporter and increasing amounts of SPI-2 (E), CrmA (F), TB13R and TB14R (G), and C7 (H). Twenty hours after transfection, the cells were treated with IFN-γ for 12 hours or left uninfected before the luciferase reporter assays.

All experiments were repeated at least three times with similar results. The bar graphs show the mean ± S.D. (n=3) of a representative experiment performed in triplicate. *p<0.05, **p<0.01, ***p<0.001, relative to the control with SeV infection.

**Figure S2. Effects of SPI-2 and CrmA on HSV-1 and VSV replication.**

SPI-2-Flag (A) and CrmA-Flag (B) stable THP-1 cells were infected with HSV-1 or VSV (MOI=0.1). The supernatants were harvested 36 hours after infection for measurements of the viral titers with standard plaque assays.

All experiments were repeated at least three times with similar results. The bar graphs show the mean ± S.D. (n=3) of a representative experiment performed in triplicate. *p<0.05, **p<0.01, ***p<0.001.

**Figure S3. Effects of SPI-2 and CrmA on VISA-mediated activation of IFN-β promoter and ISRE reporter.**

HEK293 cells were transfected with the indicated reporter and VISA together with increasing amounts of SPI-2 (A) and CrmA (B) for 20 hours before the luciferase reporter assays.

All experiments were repeated at least three times with similar results. The bar graphs show the mean ± S.D. (n=3) of a representative experiment performed in triplicate. *p<0.05, **p<0.01, ***p<0.001, relative to the control transfected with VISA.

**Supplementary Information**

Full length blots


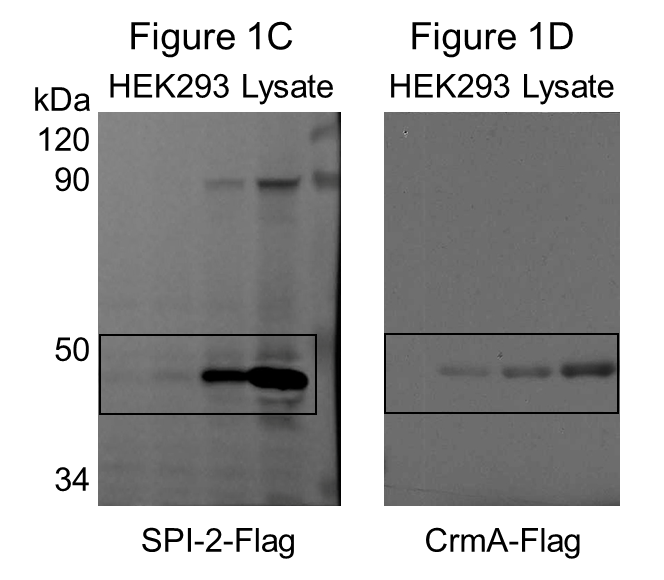


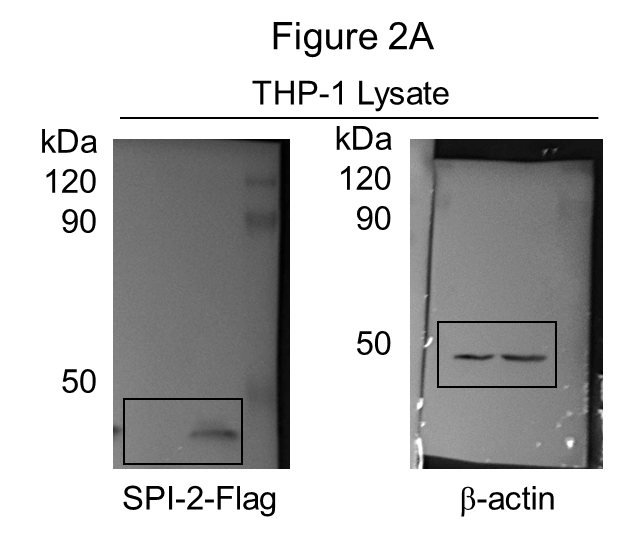


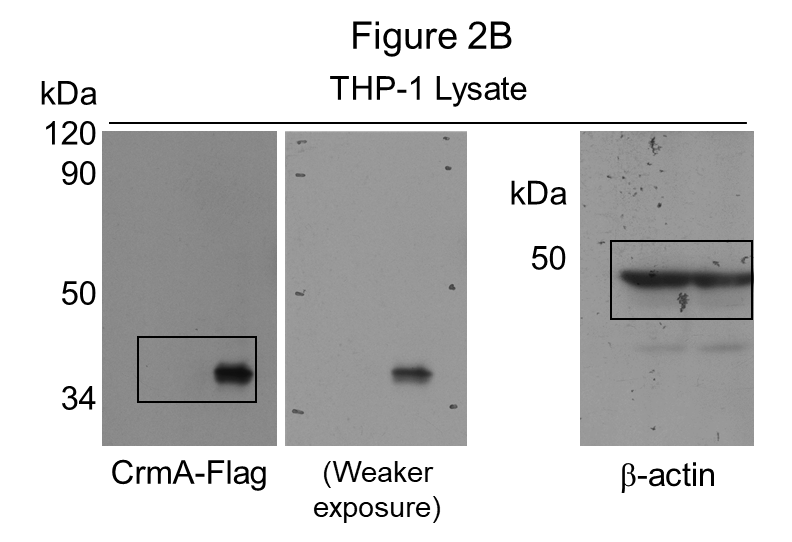


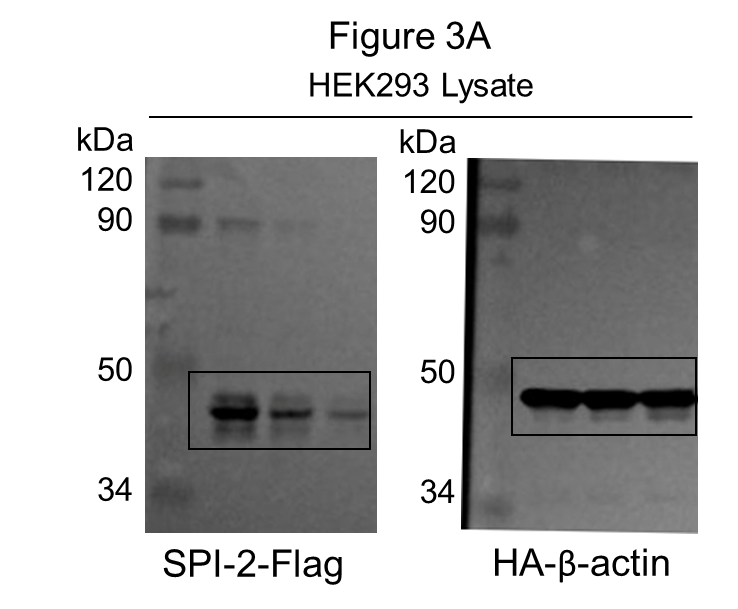


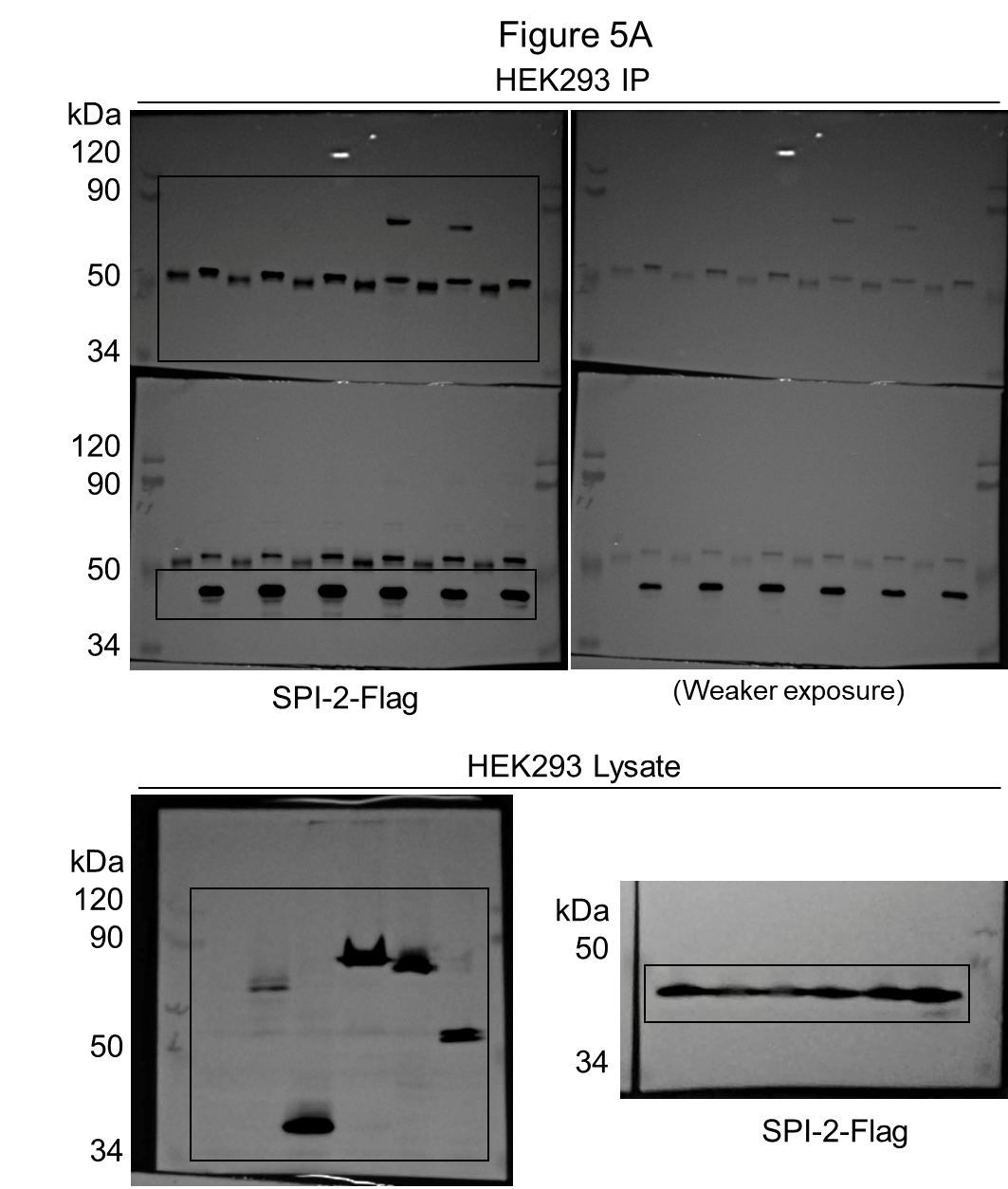


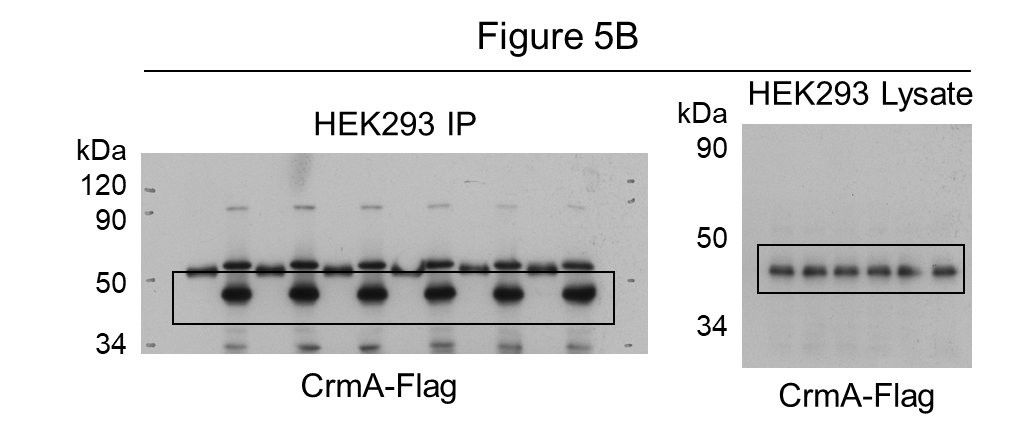


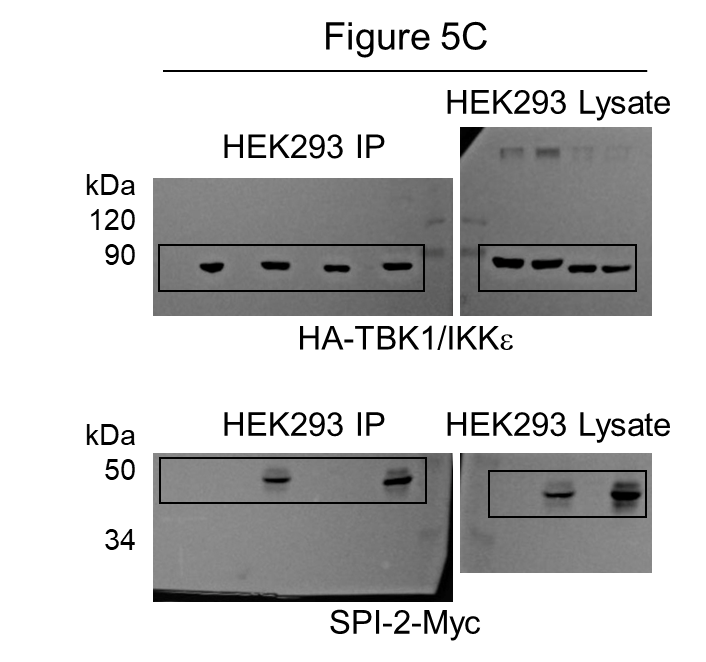


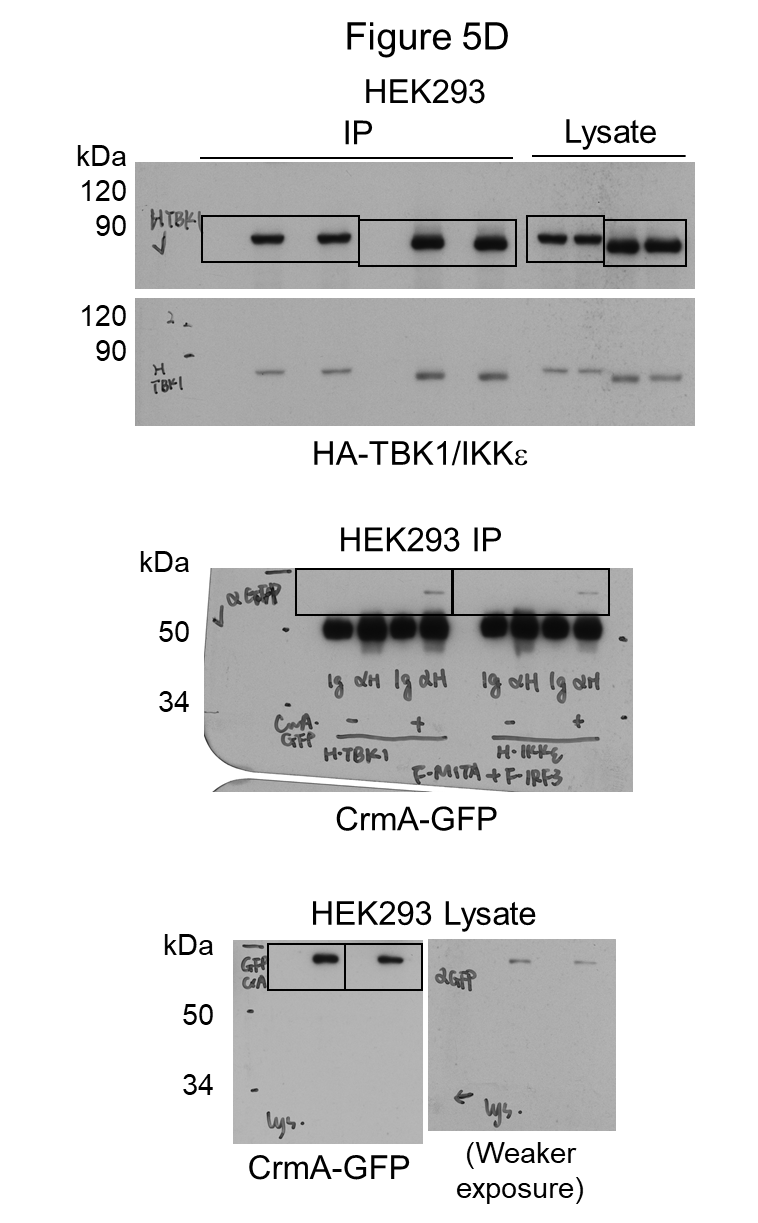

Supplement: Supplementary file 1 — Dataset 1 [file 41598_2017_11016_MOESM1_ESM.doc]
